# LAVRF: Sign language recognition via Lightweight Attentive VGG16 with Random Forest

**DOI:** 10.1371/journal.pone.0298699

**Published:** 2024-04-04

**Authors:** Edmond Li Ren Ewe, Chin Poo Lee, Kian Ming Lim, Lee Chung Kwek, Ali Alqahtani

**Affiliations:** 1 Faculty of Engineering and Technology, Multimedia University, Melaka, Malaysia; 2 Faculty of Information Science and Technology, Multimedia University, Melaka, Malaysia; 3 Department of Computer Science, King Khalid University, Abha, Saudi Arabia; 4 Center for Artificial Intelligence (CAI), King Khalid University, Abha, Saudi Arabia; Najran University College of Computer Science and Information Systems, SAUDI ARABIA

## Abstract

Sign language recognition presents significant challenges due to the intricate nature of hand gestures and the necessity to capture fine-grained details. In response to these challenges, a novel approach is proposed—Lightweight Attentive VGG16 with Random Forest (LAVRF) model. LAVRF introduces a refined adaptation of the VGG16 model integrated with attention modules, complemented by a Random Forest classifier. By streamlining the VGG16 architecture, the Lightweight Attentive VGG16 effectively manages complexity while incorporating attention mechanisms that dynamically concentrate on pertinent regions within input images, resulting in enhanced representation learning. Leveraging the Random Forest classifier provides notable benefits, including proficient handling of high-dimensional feature representations, reduction of variance and overfitting concerns, and resilience against noisy and incomplete data. Additionally, the model performance is further optimized through hyperparameter optimization, utilizing the Optuna in conjunction with hill climbing, which efficiently explores the hyperparameter space to discover optimal configurations. The proposed LAVRF model demonstrates outstanding accuracy on three datasets, achieving remarkable results of 99.98%, 99.90%, and 100% on the American Sign Language, American Sign Language with Digits, and NUS Hand Posture datasets, respectively.

## Introduction

Sign Language recognition plays a pivotal role in bridging communication gaps by automatically interpreting sign language gestures and translating them into written or spoken language. This technology is invaluable in facilitating communication between individuals who use sign language and those who may not understand it. In the realm of hand gesture recognition, there are primarily two approaches [1]: *vision-based* and *data glove-based systems*. Vision-based systems utilize cameras and computer vision algorithms to capture and interpret hand movements and gestures, making them particularly suitable for real-world applications. On the other hand, data glove-based systems involve wearable devices equipped with sensors that capture hand movements directly. These approaches have opened new possibilities for sign language recognition and communication assistance.

This paper’s primary focus centres on developing a vision-based system, aiming to enhance the accessibility and effectiveness of sign language interpretation. Existing sign language recognition methods predominantly rely on deep learning techniques, such as convolutional neural networks (CNNs), to extract meaningful features from sign language gestures. Although these CNN-based models have shown promising results, they often encounter computational complexity [2, 3] and high memory requirements [4], making real-time deployment on resource-constrained devices difficult. Attaining heightened recognition accuracy is often considered advantageous when employing a model. However, it is imperative to conscientiously address computational efficiency [5] when utilizing reduced dimensions. Additionally, some models fail to adequately capture the temporal dependencies and fine-grained details crucial for accurate sign language recognition. Therefore, there is a need for a lightweight and robust model that can achieve comparable or better performance with less computational cost.

To overcome the limitations of high computational cost and low accuracy in less controlled environments, this paper introduces LAVRF, a novel model that combines the elements of both lightweight attentive VGG16 and random forest architecture. The lightweight attentive VGG16 is a modified version of the widely used VGG16 architecture, in which the complexity is reduced by decreasing the fifth convolution block. Attention layers are also incorporated to enhance the model’s focus on informative regions within the input gestures. The lightweight attentive VGG16 excels in extracting discriminative features from sign language gestures while remaining computationally efficient. The addition of attention layers allows the model to selectively attend to relevant spatial regions, capturing fine-grained details that are crucial for accurate recognition.

In LAVRF, the random forest algorithm is employed as the classifier. Random Forest is a robust ensemble learning method renowned for its versatility in handling high-dimensional data and mitigating overfitting. By constructing multiple decision trees and combining their predictions, Random Forest provides reliable and interpretable results. Its capability to handle complex data distributions and feature interactions makes it particularly well-suited for sign language recognition tasks. Furthermore, Random Forest exhibits resilience against noisy data and outliers, making it adept at handling variations in sign language gestures caused by factors like lighting conditions or different individuals.

The key contributions of this paper are as follows:

1. The introduction of the Lightweight Attentive VGG16 reduces computational complexity while maintaining high performance in representation learning. By modifying the architecture and reducing the complexity, the model becomes more computationally efficient, making it well-suited for real-time applications and resource-limited devices.
2. The incorporation of attention layers within the Lightweight Attentive VGG16 architecture enhances the model’s ability to focus on informative regions within the input data. This attention mechanism selectively attends to the most discriminative parts of the input, enabling the model to capture fine-grained details crucial for better representation learning.
3. The utilization of Random Forest as a classifier to categorize the representations learnt by a lightweight attentive VGG16 model. Sign language recognition often faces challenges arising from variations in lighting conditions, individual differences in signing styles, and the presence of noise or outliers in the data. Random Forest, as a classifier, demonstrates resilience against noisy data and outliers. It is capable of handling these variations and anomalies, ensuring robust performance even in less controlled environments.
4. Hyperparameter optimization is conducted using Optuna, an automated hyperparameter tuning mechanism that allows for the investigation of all parameters instead of having them statically defined. This approach leads to more efficient optimization and better performance compared to manual hyperparameter tuning.

The remaining sections of the paper are organized as follows: In Section “Related Works”, a review of related work on sign language recognition is provided, categorizing the literature into traditional machine learning and deep learning approaches. Section “Sign Language Recognition with Lightweight Attentive VGG16 and Random Forest” introduces the proposed LAVRF model, which includes the Lightweight Attentive VGG16 architecture, Random Forest classifier, and hyperparameter optimization. The datasets used for evaluation are discussed in Section “Datasets”, providing an overview of their characteristics and their relevance to sign language recognition. Section “Experimental Results and Analysis” reports the experimental results, covering various aspects such as ablation study, different classifiers employed, hyperparameter optimization, and a comparison with existing works. Lastly, Section “Conclusion and Future Work” concludes the paper by summarizing the key contributions made in the study of sign language recognition.

## Related works

In recent years, the field of sign language recognition has witnessed numerous studies exploring the application of both traditional machine learning and deep learning techniques. The traditional machine learning approach involves the need for feature engineering and classification using conventional machine learning methods. On the other hand, the deep learning approach predominantly relies on the utilization of deep neural networks for representation learning and classification.

### Traditional machine learning

Sadeddine et al. (2018) [6] performed hand posture recognition in three phases: hand detection, feature extraction, and classification. In the hand detection phase, Otsu thresholding and binary operations were applied to identify the hand posture region. The feature extraction phase involved various descriptors, such as Hu’s moments, Zernike moments, Local Binary Pattern (LBP), and Generic Fourier descriptors (GFD). For classification, the system employed the Probabilistic Neural Network (PNN) and Multilayer Perceptron (MLP). The PNN, with four layers, utilized a statistical algorithm called Kernel Discriminant Analysis to compute posterior probabilities for classification. The MLP consisted of an input layer, a hidden layer with 200 neurons, and an output layer with 10 neurons for Americal Sign Language (ASL) and NUS Hand Posture datasets, and 30 neurons for the Arabic Sign Language (ArSL) dataset. The method with LBP+PNN recorded the highest accuracy of 90.41% on the ArSL dataset. For the ASL dataset, the method using LBP+PNN and LBP+GFD+PNN achieved the highest accuracy of 93.33%. Furthermore, for the NUS Hand Posture dataset, GFD+MLP as well as LBP+GFD+PNN obtained the highest accuracy of 93.33%.

The study by Gajalakshmi and Sharmila (2019) [7] employed cluster-based threshold techniques, including Otsu thresholding (OT), Ridler and Calvard thresholding (RCT), and Kittler and Illingworth thresholding (KIT), to generate binary images based on some threshold values for subsequent feature extraction. The OT method determined a threshold based on the histogram of intensity images. The RCT method utilized an iterative approach known as the isodata method to iteratively select a threshold value. The KIT method utilized iterative algorithms to find an optimal threshold by evaluating bimodal histograms based on gray-level intensity. For shape-based feature extraction, the study employed the Chain Code Histogram (CCH) method, which involved evaluating histograms based on the contour along the edge of hand signs. The chain code representation was obtained by assigning codes to the contour pixels, and the histogram was calculated based on the frequently occurring discrete values. To achieve robust and distinct descriptors for hand gesture recognition, a shape kernel was constructed based on the histogram of chain code between all hand sign images within the same category. Finally, the SVM was employed in the classification. The RCT+CCH method yielded the highest accuracy of 90% on the NUS Hand Posture Dataset.

### Deep learning

Daroya et al. (2018) [8] adopted the DenseNet architecture for sign language recognition. The network consists of four dense blocks, each comprising ten layers of batch normalization, ReLU activation, and a 3×3 convolutional layer. Three transition layers were utilized to reduce the feature map size after each dense block. The network’s output was a 24-dimensional vector representing letter probabilities. Data augmentation techniques, such as random rotations, horizontal and vertical shifting, shearing, and zooming, were utilized to increase the variety in the dataset. The proposed model achieved an accuracy of 90.3% on the ASL Dataset.

Cayamcela and Lim (2019) [9] performed transfer learning on the AlexNet and GoogLeNet for sign language recognition. AlexNet consists of eight layers, including five convolutional layers and three fully connected layers, with innovative features like ReLU activation, overlapping pooling, and dropout regularization. GoogLeNet is characterized by its inception modules that allow for efficient and parallel feature extraction at multiple scales using 1×1, 3×3, and 5×5 convolutions. The fine-tuned AlexNet and GoogLeNet achieved accuracies of 99.39% and 95.52%, respectively, when evaluated on the ASL dataset.

Likewise, Adithya and Rajesh (2020) [10] employed a CNN architecture specifically designed for gesture recognition. The architecture encompasses an input layer, three convolution layers integrated with ReLu and maxpooling layers to extract informative features, and a final fully connected output layer responsible for classifying the gestures. The proposed CNN model achieved an accuracy of 99.96% and 94.7% on the ASL Dataset and the NUS Hand Posture Dataset, respectively.

Tan et al. (2021) [11] introduced an Enhanced DenseNet (EDenseNet) that aimed to improve feature reuse through dense connectivity. This approach enhanced gradients flow and feature propagation among Convolutional (Conv) layers within dense blocks, resulting in improved recognition accuracy. EDenseNet required fewer trainable parameters while achieving comparable or superior performance compared to other CNN variants. The network architecture of EDenseNet consists of three dense blocks, each containing multiple Conv layers. Transition layers, consisting of bottleneck layers and Conv layers followed by pooling, connected the dense blocks. This modified transition layer strengthened feature propagation and generalization. Additionally, the utilization of max pooling in most transition layers further enhanced the ability to extract significant features. The EDenseNet model obtained the accuracies of 99.99%, 99.64%, and 99.30% for the ASL, ASL with Digits, and NUS Hand Posture datasets, respectively.

The study conducted by Bhaumik et al. (2022) [12] introduced a novel approach named the Lightweight Intensive Feature Extrication Deep Network (ExtriDeNet). This network architecture consists of two sequential modules: the Intensive Feature Fusion Block (IFFB) and the Intensive Feature Assimilation Block (IFAB). The primary goal of ExtriDeNet is to learn spatial characteristics of gestures from static images, with a specific emphasis on reducing the trainable parameters to lower the computational cost associated with hand gesture recognition systems. The IFFB is designed to capture rich domain knowledge related to hand postures, contributing to a comprehensive understanding of gesture spatial features. On the other hand, IFAB plays a crucial role in enhancing the network’s discriminability by preserving only the most dominant features, thus optimizing the network’s efficiency. The proposed ExtriDeNet achieved impressive results, boasting a high accuracy rate of 98.75% on the NUS dataset with inference time of 1.3 seconds.

In 2023, Bhaumik et al. [13] proposed the Hybrid Feature Attention Network (HyFiNet) as a solution for hand gesture recognition, achieving notable success with a high accuracy of 97.78% on the NUS dataset. The network’s architecture is characterized by the sequential stacking of four multi-scale refined edge extraction modules. These modules are strategically designed to capture refined edge information, and this is further enhanced by the incorporation of a hybrid feature attention block. The HyFiNet achieved an accuracy of 97.78% and inference time of 6.4 seconds on the NUS dataset.

Tan et al. (2023) [14] introduced HGR-ViT, a Vision Transformer (ViT) model incorporating an attention mechanism designed for hand gesture recognition. The approach began by dividing a given hand gesture image into fixed-size patches. These patches were then enriched with positional embedding, creating learnable vectors that encapsulate the positional information of the hand patches. The resulting sequence of vectors served as the input to a standard Transformer encoder, producing a representation of the hand gesture. To classify the hand gesture into the correct class, a MLP head was added to the output of the encoder. HGR-ViT demonstrated impressive accuracy across three benchmark datasets: 99.98% for the ASL dataset, 99.36% for the ASL with Digits dataset, and 99.85% for the NUS Hand Posture dataset.

Later, Tan et al. (2023) [15] presented another novel approach, named Stacked Distilled Vision Transformers (SDViT), for hand gesture recognition. The methodology involved the stacking of distilled Vision Transformers (ViTs) to enhance predictive performance and mitigate overfitting. Initially, a pretrained ViT, incorporating a self-attention mechanism, was introduced to adeptly capture intricate connections among image patches. This mechanism proves beneficial in addressing the challenge posed by the high similarity between hand gestures. Subsequent to the ViT’s training, knowledge distillation was applied to compress the model, thereby improving its generalization. The innovative aspect of SDViT lies in the stacking of multiple distilled ViTs, resulting in a robust and high-performing model. The proposed SDViT achieved exemplary accuracy of 100.00% on the ASL dataset, 99.60% on the ASL with digits dataset, and 100.00% on the NUS Hand Posture dataset.

Likewise, Zhang et al. (2023) [5] employed ViT for static hand gesture recognition. The pivotal aspect of the proposed architecture centers around the utilization of a multi-head attention mechanism, which focuses on learning global feature information. This mechanism comprises a linear layer, self-attention layer, concatenation layer (for combining the outputs of multiple attention heads), and a final linear layer. The reported accuracy was 96.43% for the ASL dataset.

Gupta et al. (2023) [16] amalgamated ViT and a lightweight CNN for hand gesture recognition. The model incorporated a conventional ViT structure alongside a lightweight CNN architecture, comprising three convolution layers, two bottleneck layers, and a batch normalization layer. The primary emphasis of the lightweight CNN is on Depth Squeezing and Stretching, strategically employed to reduce the input size and conserve computational resources effectively. Notably, the architecture achieved an accuracy of 98.17% on the NUS dataset and an even higher accuracy of 98.7% on the ASL dataset. The summary of sign language recognition methods and its accuracy achieved is shown in Table 1.

**Table 1:** Summary of sign language recognition methods.

Method	Dataset	Accuracy (%)
LBP + PNN [6]	ArSL, ASL, NUS Hand Posture	90.41, 93.33, 93.33
RCT + CCH + SVM [7]	NUS Hand Posture	90
DenseNet [8]	ASL Dataset	90.30
AlexNet, GoogLeNet [9]	ASL Dataset	99.39, 95.52
CNN [10]	ASL Dataset, NUS Hand Posture Dataset	99.96, 94.7
Enhanced DenseNet (EDenseNet) [11]	ASL, ASL with Digits, NUS Hand Posture Dataset	99.99, 99.64, 99.30
ExtriDeNet [12]	NUS Hand Posture Dataset	98.75
HyFiNet [13]	NUS Hand Posture Dataset	97.78
ViT [14]	ASL, ASL with Digits, NUS Hand Posture Dataset	99.98, 99.36, 99.85
SDViT [15]	ASL, ASL with Digits, NUS Hand Posture Dataset	100, 99.60, 100
ViT [5]	ASL	96.43
ViT + Lightweight CNN [16]	ASL, NUS Hand Posture Dataset	98.17, 98.7

The existing sign language recognition techniques typically rely on manually engineered features and traditional machine learning algorithms for classification. Recent approaches have leveraged deep learning models for both feature extraction and classification. While traditional machine learning algorithms excel at classification, deep learning models are proficient in representation learning. To combine the strengths of both, this paper presents a hybrid model that utilizes a lightweight attentive VGG16 network for representation learning and ensemble learning algorithm for classification. By combining multiple models, each with its own strengths and weaknesses, this hybrid approach can significantly enhance the accuracy of sign language recognition. However, hyperparameters can significantly affect the performance of the model, and fine-tuning the model is essential to obtain the best results. The next section provides details on the proposed hybrid model for sign language recognition and the strategies used for hyperparameter optimization.

## Sign language recognition with Lightweight Attentive VGG16 and Random Forest

The proposed method, called Lightweight Attentive VGG16 with Random Forest (LAVRF), combines a streamlined variant of the VGG16 model with an attention mechanism and employs a Random Forest classifier for sign language recognition. The Lightweight Attentive VGG16 architecture reduces complexity while incorporating attention modules that dynamically focus on relevant regions of input images, capturing fine-grained details and improving recognition accuracy.

**Fig 1:**
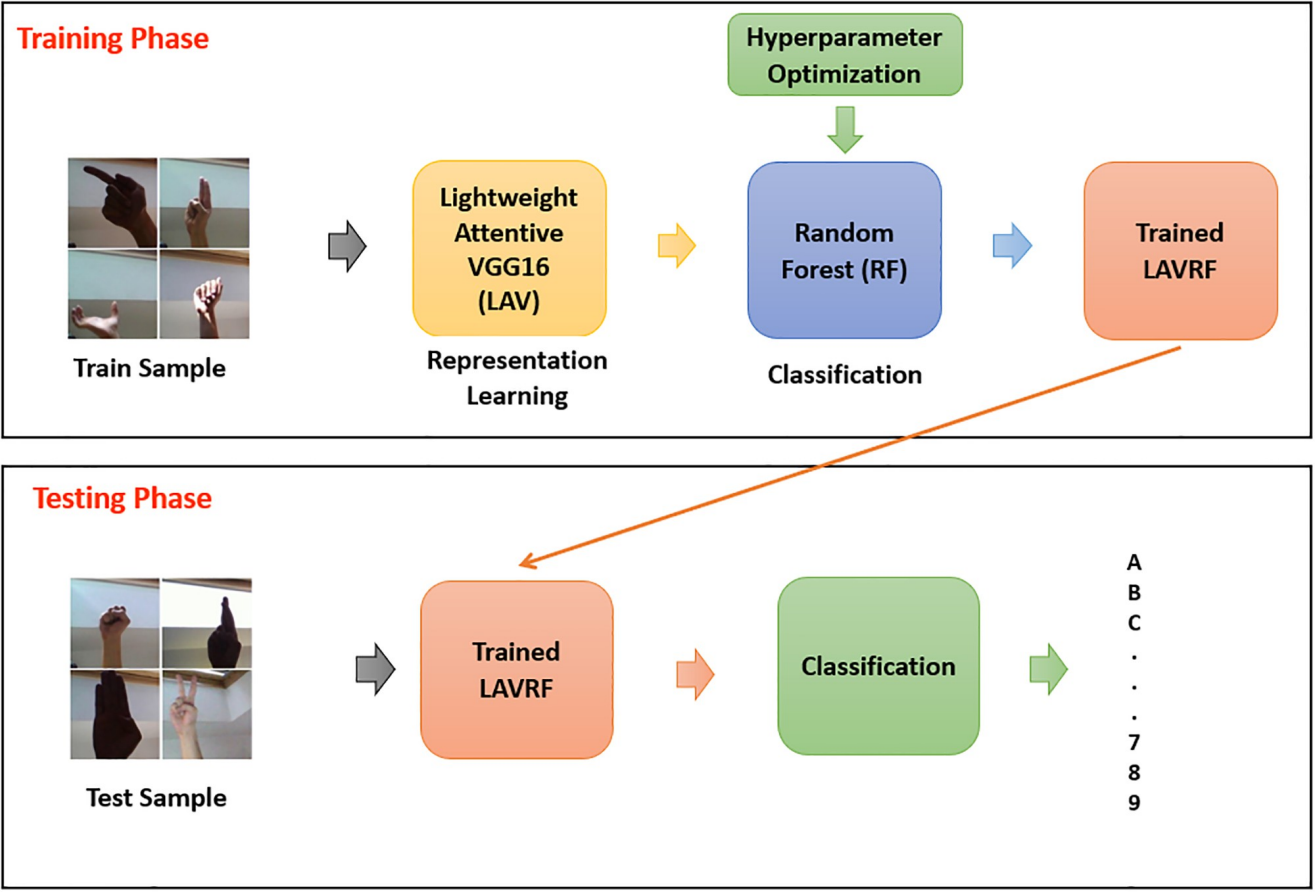
Process flow of sign language recognition using LAVRF model.

Using a Random Forest classifier offers several advantages. Firstly, Random Forest handles high-dimensional feature representations effectively, enabling the model to learn complex decision boundaries in the rich feature space of the Lightweight Attentive VGG16. Secondly, the ensemble nature of Random Forest reduces variance and overfitting, enhancing robustness and generalization. This is crucial in sign language recognition, where the model needs to perform well on unseen gestures and variations. The process flow of the sign language recognition with LAVRF is illustrated in Fig 1.

### Representation learning with Lightweight Attentive VGG16

**Fig 2:**
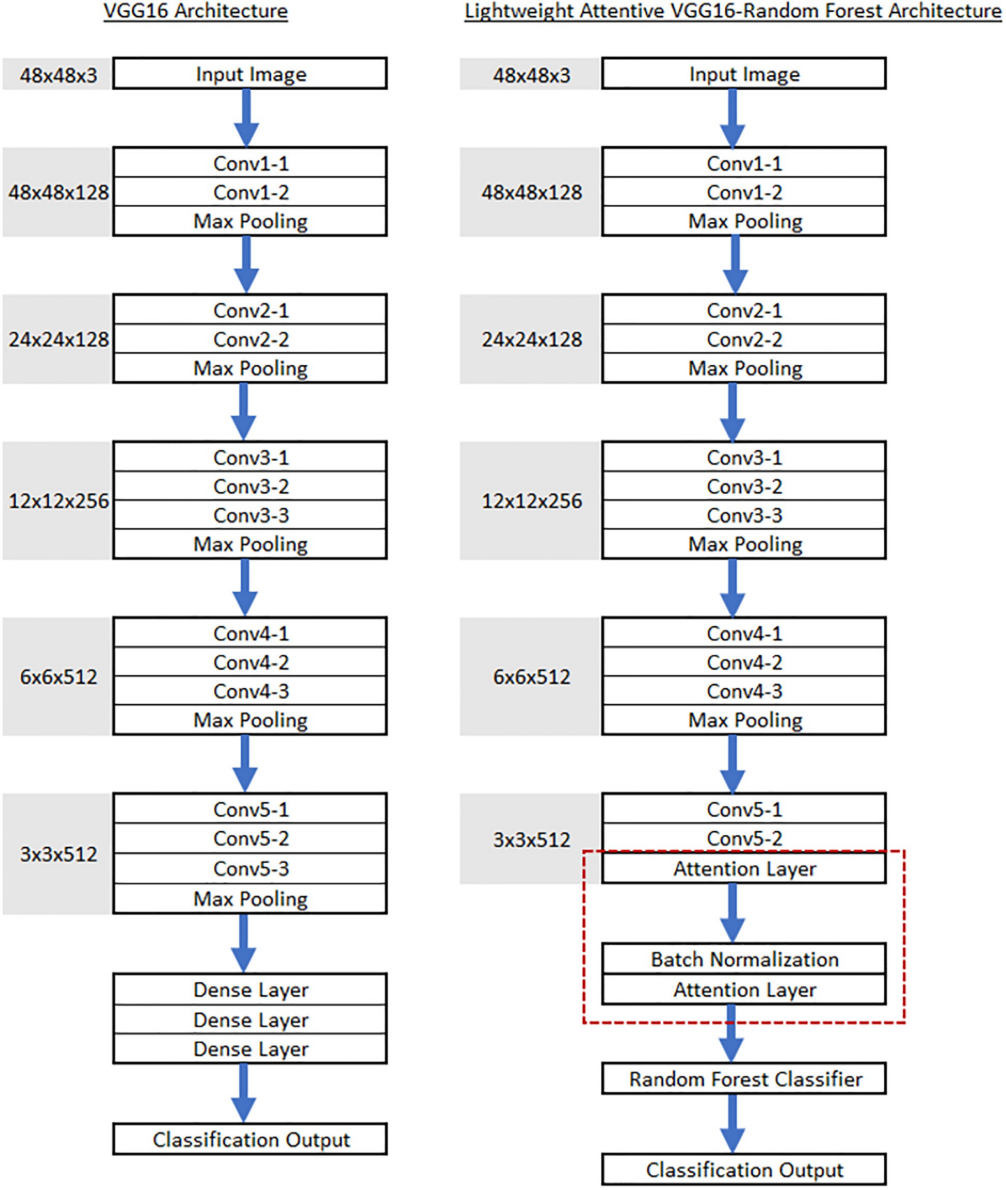
Comparison of VGG16 and Lightweight Attentive VGG16 architecture.

The proposed architecture, known as the Lightweight Attentive VGG16 model, is a streamlined variant of the VGG16 model, designed to reduce complexity while incorporating an attention mechanism. A side-by-side comparison of the original VGG16 and the Lightweight Attentive VGG16 architectures is provided in Fig 2. Compared to the original VGG16, the Lightweight Attentive VGG16 model preserves the initial four convolution blocks, consisting of multiple convolutional layers followed by a max-pooling layer. These convolutional layers utilize 3×3 filters with a stride of 1 and padding of 1, effectively preserving spatial information. The subsequent max-pooling layers perform 2×2 pooling with a stride of 2, downsampling the feature maps to retain essential features. The fifth convolution block is modified, with the third convolutional layer, max-pooling layer, and subsequent fully connected layers replaced by an attention layer, followed by a batch normalization layer, and another attention layer.

In the proposed model architecture, attention layers are strategically incorporated to optimize feature representation and enhance the overall efficiency of the CNN. The initial attention layer is positioned immediately after the convolutional layer. To optimize the attention layer’s effectiveness, scaled dot-product attention [17] is employed. This attention mechanism involves three matrices: the query matrix (*Q*), key matrix (*K*), and value matrix (*V*). These matrices are obtained by projecting the output from the previous convolutional layer *X* into different spaces using learnable weight matrices. Mathematically, *Q* = *X* ⋅ *W*_*Q*_, *K* = *X* ⋅ *W*_*K*_, and *V* = *X* ⋅ *W*_*V*_, where *W*_*Q*_, *W*_*K*_, and *W*_*V*_ are the respective learnable weight matrices. The attention mechanism formula is then applied using these matrices:

$$\text{Attention}\left(X\right)=\text{softmax}(\frac{QK^{T}}{\sqrt{d_{k}}})V$$

Here, softmax is applied element-wise, and *T* denotes the transpose. The scaling factor $\sqrt{d_{k}}$ is crucial to prevent the dot products from becoming too large, contributing to the stability and regularization of the learning process. This mechanism allows the model to selectively emphasize certain features based on their relevance, improving the network’s ability to capture intricate patterns and spatial dependencies in the input data.

Following the first attention layer, batch normalization is introduced to normalize the emphasized features, offering stability to the learning process, and mitigating internal covariate shift issues. This normalization step contributes to the overall efficiency and effectiveness of the network during the training phase. To further refine contextual information, another attention layer is placed after batch normalization. This additional attention mechanism enables the model to adaptively adjust the importance of features based on their interdependencies.

The dual attention layers not only enhance the model’s discriminative capabilities but also serve as a regularization mechanism [18]. By focusing on relevant features and learning adaptive weights through scaled dot-product attention, the attention mechanism helps prevent overfitting, ensuring that the model generalizes well to unseen data. This strategic integration of attention mechanisms, especially with scaled dot-product attention, underscores the model’s efficiency, stability, and capacity to capture both local and global contextual information [13] for improved performance in diverse computer vision tasks.

Importantly, the proposed lightweight attentive VGG16 architecture significantly reduces the number of parameters from 14,977,857 in the original VGG16 to 10,017,611, enabling faster processing times while still benefiting from ImageNet training. The architectural details of the Lightweight Attentive VGG16 are presented in Table 2.

**Table 2:** Layer-wise architecture of the proposed Lightweight Attentive VGG16 model.

Model	Layers	Configurations
Lightweight VGG-16	Conv 1-1	3 × 3 conv, stride = 1, padding = 1
	Conv 1-2	3 × 3 conv, stride = 1, padding = 1
	Max-Pooling	2 × 2, stride = 2
	Conv 2-1	3 × 3 conv, stride = 1, padding = 1
	Conv 2-2	3 × 3 conv, stride = 1, padding = 1
	Max-Pooling	2 × 2, stride = 2
	Conv 3-1	3 × 3 conv, stride = 1, padding = 1
	Conv 3-2	3 × 3 conv, stride = 1, padding = 1
	Conv 3-3	3 × 3 conv, stride = 1, padding = 1
	Max-Pooling	2 × 2, stride = 2
	Conv 4-1	3 × 3 conv, stride = 1, padding = 1
	Conv 4-2	3 × 3 conv, stride = 1, padding = 1
	Conv 4-3	3 × 3 conv, stride = 1, padding = 1
	Max-Pooling	2 × 2, stride = 2
	Conv 5-1	3 × 3 conv, stride = 1, padding = 1
	Conv 5-2	3 × 3 conv, stride = 1, padding = 1
Attention	Attention	score mode = dot
	Batch Normalization	momentum = 0.99, epsilon = 0.001
	Attention	score mode = dot

### Classification with Random Forest

The utilization of a Random Forest classifier to categorize representations acquired by the Lightweight Attentive VGG16 model in sign language recognition presents numerous benefits. Random Forest, an ensemble learning method, combines multiple decision trees for predictions, proving advantageous in handling high-dimensional data. The Lightweight Attentive VGG16 model generates high-dimensional feature representations, with each dimension corresponding to specific aspects of the input image. Random Forest adeptly manages this feature space, effectively discerning complex decision boundaries for class separation. Its ensemble nature addresses overfitting concerns, reducing variance and enhancing robustness, crucial in sign language recognition for effective generalization. This classifier helps mitigate overfitting risks and bolsters generalization. Additionally, Random Forest’s resilience to noisy and incomplete data aligns with real-world challenges encountered in sign language recognition systems, such as lighting variations or occlusions.

### Hyperparameter optimization of Random Forest

Hyperparameter optimization is a crucial step in building machine learning models that involves setting the right hyperparameters for optimal performance. However, finding the right hyperparameters can be challenging and time-consuming, requiring a deep understanding of the model’s hyperparameters and their relationships. In this study, Optuna [19] with hill climbing is leveraged for hyperparameter optimization. Optuna provides a flexible and efficient framework for automating the search process, enabling the discovery of optimal hyperparameter configurations.

Optuna employs a Tree-structured Parzen Estimator (TPE) algorithm, which dynamically allocates computational resources to promising hyperparameter settings, leading to faster convergence and improved performance. The TPE algorithm is a sequential model-based optimization (SMBO) technique commonly employed for hyperparameter optimization. In this context, the objective function *f*(*θ*) represents the performance metric (e.g., accuracy) of the Random Forest with hyperparameters *θ* such as the number of trees, maximum depth, and minimum samples per leaf. The TPE algorithm proceeds through iterations, initializing with an initial set of hyperparameter configurations. It models the conditional probability distributions of “good” and “bad” configurations based on observed outcomes. The algorithm then samples new configurations from these distributions, evaluates their performance, and updates the probability distributions accordingly. This iterative process guides the search toward optimal hyperparameter configurations for the Random Forest.

The integration of hill climbing within Optuna further enhances the efficiency and effectiveness of the hyperparameter optimization process. Hill climbing is a local search optimization algorithm that can be applied within the Optuna framework. In the context of hyperparameter optimization, the objective is to find the configuration of hyperparameters that minimizes or maximizes a given objective function. Hill climbing iteratively explores the neighborhood of the current hyperparameter configuration and updates the configuration based on the improvement in the objective function.

Given the hyperparameter configuration of Random Forest *θ* and the objective function *f*(*θ*), the hill climbing algorithm works as follows. Starting from an initial configuration, it evaluates the objective function. It then explores nearby configurations by making small adjustments to the hyperparameters. If a neighboring configuration leads to a better objective function value, the algorithm moves to that configuration and repeats the process. The iteration continues until no further improvement is possible in the local neighborhood. The hill climbing update rule can be expressed as:

$$\theta_{\text{new}}=\theta_{\text{current}}+\Delta \theta$$

where Δ*θ* represents the change in hyperparameters. The choice of Δ*θ* is crucial, as it determines the step size in the search space. The algorithm stops when no improvement is observed in the objective function or after a predefined number of iterations. This iterative process helps to narrow down the search space and converge to more optimal hyperparameter configurations for sign language recognition.

## Datasets

This section provides an overview of the datasets used for performance evaluation: the ASL dataset [20], the ASL with Digits dataset [21], and the NUS Hand Posture dataset [22].

### American Sign Language (ASL) dataset

**Fig 3:**
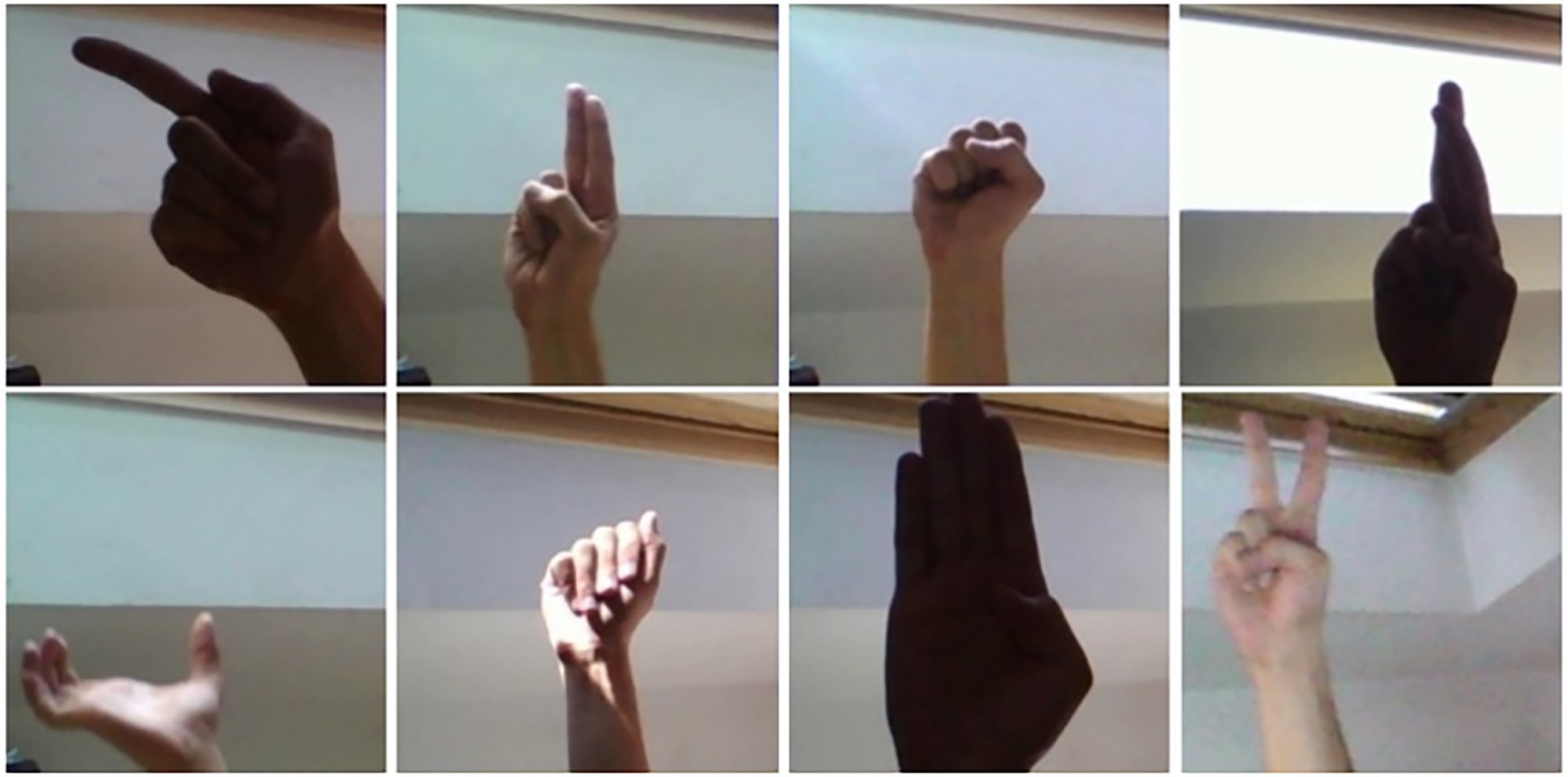
Sample images from ASL dataset.

The American Sign Language (ASL) dataset is a collection of 24 classes of alphabet gestures (A to Y). Each class of gesture is signed by five signers. The alphabet J and Z are excluded from the dataset as they are dynamic gestures. The dataset comprises a total of 65,774 images with a resolution of 200×200 pixels. Fig 3 shows some sample images from ASL Dataset.

### ASL with Digits Dataset

**Fig 4:**
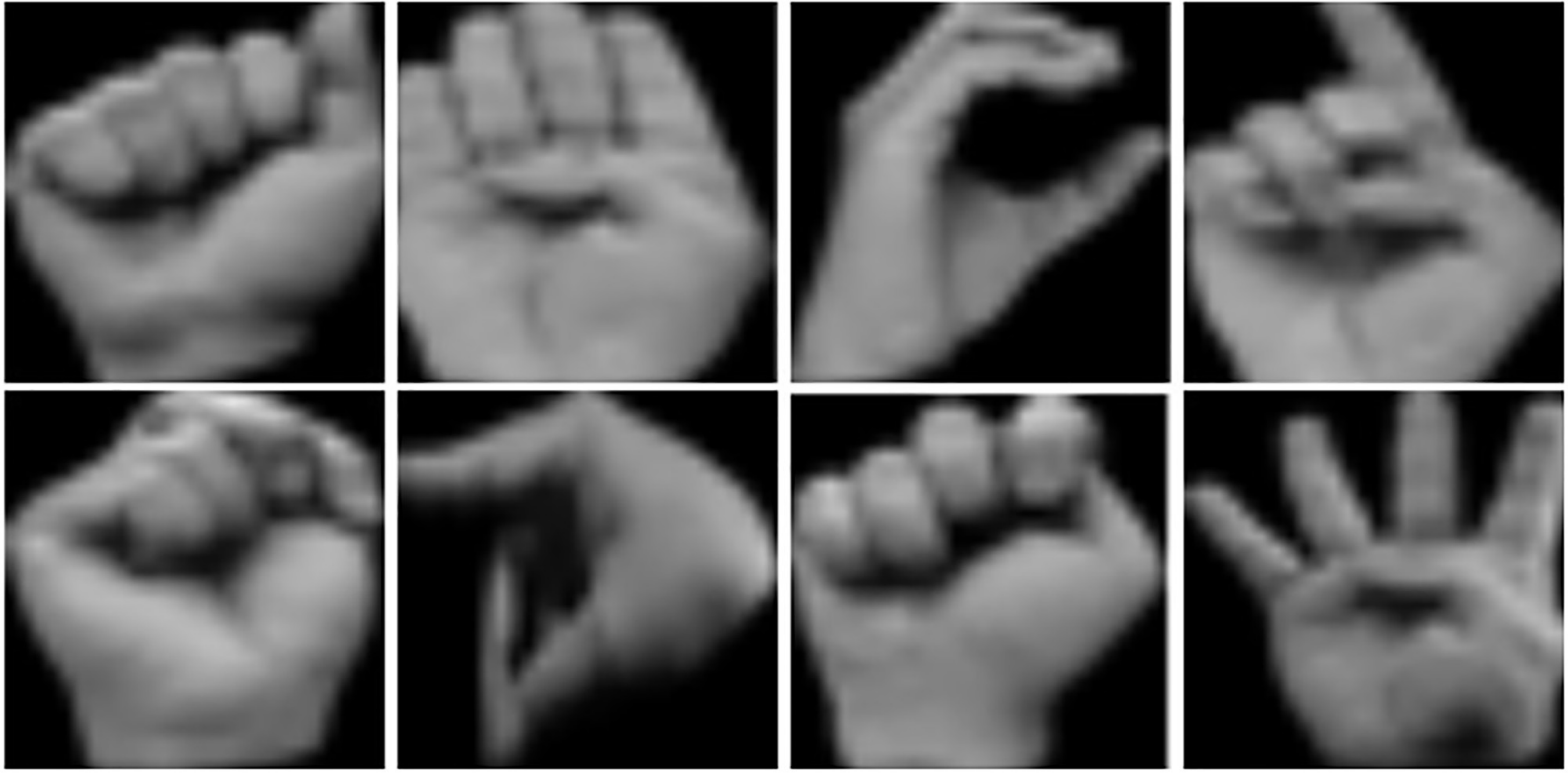
Sample images from ASL with Digits Dataset.

The ASL with Digits Dataset comprises a total of 36 classes, including 26 for alphabets (A to Z) and 10 for digits (0 to 9). The dataset is signed by five proficient signers, encompassing a total of 2,515 images. Fig 4 shows some sample images from ASL with Digits Dataset.

### NUS Hand Posture Dataset

**Fig 5:**
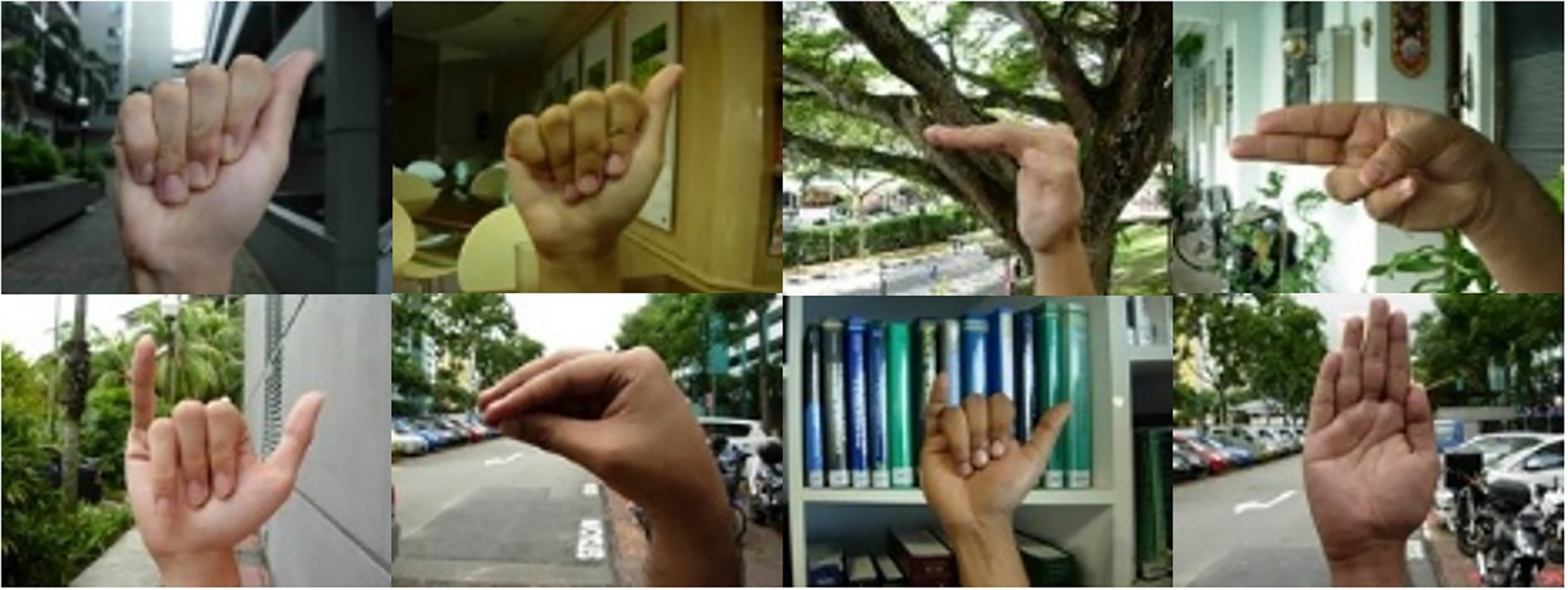
Sample images from NUS Hand Posture Dataset.

The NUS Hand Posture Dataset consists of 10 classes of hand postures, which were obtained by varying the placement and size of the hand in the camera frame. The dataset captures a diverse range of backgrounds and hands and was collected at the National University of Singapore (NUS). The hand poses were performed by 40 individuals from various nationalities and origins. This dataset comprises a total of 2,000 images. Fig 5 shows some sample images from NUS Hand Posture Dataset. The summary of the datasets is provided in Table 3.

**Table 3:** Summary of datasets.

Dataset	Num of Class	Total Samples
ASL	24	65,774
ASL with Digits	36	2,515
NUS Hand Posture	10	2,000

## Experimental results and analysis

This section presents an ablation study of the LAVRF model to showcase the individual contributions and significance of each enhancement. The experimental results also include the evaluation of LAVRF with various ensemble classifiers. To attain optimal performance, hyperparameter optimization is conducted using Optuna with a TPE and hill climbing approach. Furthermore, a comparison of the results with existing works in sign language recognition is provided. In the experiments, the Adam optimizer with a learning rate of 0.0001, categorical cross-entropy as the loss function, and accuracy as the evaluation metric are utilized. In addition, early stopping with training loss as the monitoring metric and a patience of 20 is employed.

### Ablation study of LAVRF

This subsection describes the ablation study conducted on the NUS Hand Posture Dataset. The NUS Hand Posture Dataset was chosen for experimentation due to its smaller size and more complex background compared to the other evaluated datasets. The ablation study demonstrates the progressive performance enhancements achieved by incorporating various components, namely lightweight VGG16 variants, attention mechanisms, and Random Forest classifier into the sign language recognition model.

As shown in Table 4, the original VGG16 model achieved an average accuracy of 80.85% with an inference time of 2.54 seconds. The introduction of the Lightweight VGG16 variant resulted in an enhanced average accuracy of 84.50%, while significantly reducing the inference time to a mere 0.02 seconds. This improvement can be attributed to the optimization and streamlining of the VGG16 architecture, enabling faster and more efficient inference. Further refinement in the form of the Lightweight Attentive VGG16 model yielded a higher average accuracy of 84.95%, with a slight increase in the inference time to 0.08 seconds. The addition of attention mechanisms likely contributed to the improved accuracy by allowing the model to focus on relevant features and enhance its ability to distinguish between different hand postures.

**Table 4:** Results of ablation studies on the NUS Hand Posture Dataset.

Classifier Model	Average Accuracy (%)	Inference Time (s)
VGG16	80.85	2.54
Lightweight VGG16	84.50	0.02
Lightweight Attentive VGG16	84.95	0.08
Lightweight Attentive VGG16 with Random Forest (LAVRF)	95.25	0.008

The proposed LAVRF model, achieved the highest average accuracy of 95.25%, while consuming an incredibly low inference time of 0.008 seconds. This substantial increase in accuracy can be attributed to the integration of a Random Forest classifier, which leverages an ensemble of decision trees to further enhance the model’s predictive capabilities. The experimental results demonstrated that the introduction of lightweight variants, attention mechanisms, and Random Forest classifier have collectively led to significant improvements in average accuracy and inference time in sign language recognition.

### Experimental results of different classifiers

This subsection presents the experimental results of different classifiers using the representation learned by the Lightweight Attentive VGG16 model on the NUS Hand Posture Dataset. The evaluated classifiers include Random Forest, Extra Trees, Light Gradient Boosting Machine (LightGBM), Extreme Gradient Boosting (XGBoost), and Categorical Boosting (CatBoost).

Extra Trees classifier is a type of ensemble learning technique that combines the results of multiple de-correlated decision trees to produce its classification result. In contrast to Random Forest, which uses bootstrapped replicas, Extra Trees uses the entire original sample for training each decision tree. During the classification process, each decision tree in the Extra Trees model is provided with a random sample of *k* features from the total set of features. This random selection of features helps to create multiple de-correlated decision trees and reduces the risk of overfitting. In comparison to Random Forest, Extra Trees is faster in terms of execution time as it randomly chooses split nodes instead of optimizing the split like Random Forest.

LightGBM is a gradient boosting algorithm that utilizes decision trees to improve model efficiency and reduce memory usage. It uses unique techniques like Gradient-based One Side Sampling (GOSS) and Exclusive Feature Bundling (EFB) to overcome the limitations of histogram-based algorithms commonly used in gradient boosting decision tree (GBDT) frameworks. GOSS helps to retain the accuracy of information gain estimation by only randomly dropping instances with small gradients while retaining those instances with large gradients. EFB, on the other hand, is a near-lossless method of reducing the number of effective features by exploiting the fact that many features in sparse feature spaces are nearly exclusive, meaning they rarely take non-zero values at the same time. These innovative techniques allow LightGBM to effectively learn from large datasets and make accurate predictions, making it a popular choice for machine learning tasks.

XGBoost is a powerful and widely used gradient boosting library that offers high efficiency, flexibility, and portability. The algorithm uses a step-by-step forward additive model with a decision tree foundation. XGBoost builds trees by continuously splitting the features and fitting the residuals from the regression prediction of the previous *t*-1 tree when forming the *t*-th tree. The tree structure is optimized by minimizing the value of the objective function, until *T* trees are obtained. The prediction for each sample is calculated by averaging the scores assigned to each tree, where each leaf node corresponds to a score. In addition to its highly optimized gradient boosting framework, XGBoost also implements machine learning algorithms that improve the training time by using parallel tree boosting. It trains thousands of models on different subsets of the training data and selects the top model through voting. To further prevent overfitting, XGBoost employs regularization penalties. All these features make XGBoost a reliable and efficient tool for a wide range of machine learning tasks.

CatBoost is a GBDT machine learning algorithm that stands out from other GBDTs due to its efficient handling of categorical features and ordered boosting. The algorithm builds a set of decision trees consecutively, where each tree is built with reduced loss compared to the previous trees, effectively boosting the performance of the model. One of the key features of the CatBoost algorithm is its ability to handle categorical features effectively. Conventional GBDTs struggle to interpret categorical features and therefore rely on techniques like one-hot encoding or gradient statistics to transform them into useful information. However, this can result in large memory and computational resource requirements, especially for categorical features with high repeatability. The CatBoost algorithm addresses this by using efficient modified target-based statistics to handle categorical features during training, saving considerable computational time and resources. Another key aspect of the CatBoost algorithm is its ordered boosting mechanism. Conventional GBDTs can result in a prediction shift in the developed model, leading to a unique target leakage issue, after performing several boosting steps on all the training data. The CatBoost algorithm avoids this by employing ordered boosting, a permutation-driven strategy, to train the model on one piece of data while computing residuals on a different subset of data. This prevents overfitting and ensures the reliability of the developed model.

The findings in Table 5 indicate that Random Forest achieves the highest average accuracy of 95.25% among the five classifiers. Extra Trees follows with an average accuracy of 94.00%. CatBoost, LightGBM, and XGBoost exhibit lower average accuracies of 93.80%, 93.05%, and 92.05%, respectively. The use of the lightweight VGG16 model with attention layers allows for the extraction of rich and expressive features from the hand posture images, resulting in a high-dimensional feature space. Random Forest’s ensemble learning approach, which combines multiple decision trees, enables the capturing of complex relationships and interactions within these learned features. The aggregation of predictions from multiple trees in Random Forest mitigates the risk of overfitting and improves generalization to unseen data. Furthermore, Random Forest demonstrates robustness to noise and outliers through the utilization of random feature subsets and bootstrapping during training. This robustness makes it well-suited for effectively handling the variations in hand postures and background conditions present in the NUS Hand Posture Dataset, thus facilitating accurate classification.

**Table 5:** Experimental results of different classifiers on the NUS Hand Posture Dataset.

Model	Accuracy (%)						Inference Time (s)
	Fold-1	Fold-2	Fold-3	Fold-4	Fold-5	Average	
Random Forest	95.50	95.25	93.50	96.00	96.00	95.25	0.008
Extra Trees	94.75	95.00	92.00	94.25	94.00	94.00	0.007
LightGBM	95.00	94.25	91.00	91.75	93.25	93.05	0.002
XGBoost	94.25	92.50	90.75	89.75	93.00	92.05	0.004
CatBoost	96.00	94.00	91.75	93.00	94.25	93.80	0.01

### Experimental results of hyperparameter optimization

This subsection describes the experimental results of hyperparameter optimization for the Random Forest algorithm. Table 6 displays the average accuracy of 5-fold cross validation achieved on three different datasets before and after applying hyperparameter optimization. For the ASL dataset, the Random Forest achieved an average accuracy of 99.96% without hyperparameter optimization. However, with hyperparameter optimization, the accuracy improved to 99.98%. Similarly, for the ASL with Digits Dataset, the accuracy increased from 98.99% to 99.90% after hyperparameter optimization. Notably, for the NUS Hand Posture Dataset, the Random Forest achieved a perfect accuracy of 100% after hyperparameter optimization, compared to an accuracy of 95.25% without optimization. Table 7 summarizes the optimal hyperparameter settings obtained after the optimization process for each dataset.

**Table 6:** Experimental results of hyperparameter optimization on the Random Forest.

Category	Average Accuracy (%)		
	ASL	ASL with Digits	NUS Hand Posture
Without hyperparameter optimization	99.96	98.99	95.25
With hyperparameter optimization	99.98	99.90	100

**Table 7:** Summary of optimal hyperparameter settings of Random Forest.

Dataset	Optimal Hyperparameter Settings
ASL Dataset	n_jobs: -1
	criterion: gini
	max_features: 0.6
	min_samples_split: 50
	max_depth: 6
	eval_metric_name: accuracy
	num_class: 24
ASL with Digits	n_jobs: -1
	criterion: entropy
	max_features: 0.6
	min_samples_split: 50
	max_depth: 6
	eval_metric_name: accuracy
	num_class: 36
NUS Hand Posture	n_jobs: -1
	criterion: entropy
	max_features: 0.6
	min_samples_split: 50
	max_depth: 6
	eval_metric_name: accuracy
	num_class: 10

### Comparative results with the existing works

This section presents a comparative analysis of existing works in sign language recognition, focusing on their performance in Table 8. The table provides a comprehensive performance comparison of various methods across three distinct datasets: ASL, ASL with Digits, and NUS Hand Posture.

**Table 8:** Performance comparison with existing works.

Method	Average Accuracy (%)		
	ASL	ASL with Digits	NUS Hand Posture
LBP+PNN [6]	93.33	-	93.33
RCT+CCH+SVM [7]	-	-	90.00
CNN [23]	99.85	98.69	89.15
ADCNN [24]	98.50	98.49	83.10
DenseNet [8]	90.3	-	-
AlexNet [9]	99.39	-	-
GoogLeNet [9]	95.52	-	-
CNN [10]	99.96	-	94.70
EDenseNet [11]	99.99	99.64	99.30
CNN [25]	99.78	98.65	89.30
DAG-CNN [26]	99.89	98.13	91.05
ExtriDeNet [12]	-	-	98.75
HyFiNet [13]	-	-	97.78
ViT [14]	99.98	99.36	99.85
SDViT [15]	100.00	99.60	100.00
ViT [5]	96.43	-	-
ViT + Lightweight CNN [16]	98.17	-	98.7
**LAVRF (proposed)**	**99.98**	**99.90**	**100.00**

For the ASL dataset, the proposed LAVRF method achieves an outstanding accuracy of 99.98%, comparable to the EDenseNet, SDViT and ViT by Tan et al. approach as the top performers. These models outshine other methods, including CNN (99.85%), CNN by Ahuja et al. (99.78%), AlexNet (99.39%), GoogLeNet (95.52%), ADCNN (98.50%), LBP + PNN (93.33%), DAG-CNN (99.89%), ViT by Zhang et al. (96.43%) and ViT + CNN (98.7%).

The ASL with Digits dataset presents a challenge due to its limited training samples (2,515) across 36 classes. However, LAVRF demonstrates remarkable accuracy, achieving 99.90%, closely followed by EDenseNet at 99.64% and SDViT at 99.60%. LAVRF surpasses CNN (98.69%), CNN by Ahuja et al. (98.65%), ADCNN (98.49%), and DAG-CNN (98.13%) in this dataset.

Recognizing hand postures accurately in the NUS Hand Posture dataset is challenging due to its complex background. In this context, LAVRF shines with a perfect average accuracy of 100.00% matching SDViT (100.00%), showcasing its exceptional recognition capabilities. EDenseNet and ViT by Tan et al. also delivers strong performance with an accuracy of 99.30% and 99.85% respectively. Other methods, such as DAG-CNN (91.05%), CNN by Ahuja et al. (89.30%), CNN (89.15%), RCT+CCH+SVM (90.00%), LBP + PNN (93.33%), ExtriDeNet (98.75%) and HyFiNet (97.78%), exhibit varying levels of performance on this dataset.

LAVRF incorporates several modifications to the original VGG16 architecture, resulting in enhanced performance. Notably, it removes the last few layers of VGG16, reducing model complexity and addressing overfitting concerns. Additionally, attention layers are introduced to the lightweight VGG16 architecture, enabling effective representation learning. These attention layers allow the model to focus on salient features and relevant regions within the input data, capturing more discriminative information. Furthermore, LAVRF employs Random Forest as the classifier, renowned for its robustness in handling high-dimensional data and noise. The integration of Optuna with hill climbing optimizes the hyperparameters of the Random Forest classifier, enabling the classifier to adapt its decision boundaries and improve generalization capabilities.

**Fig 6:**
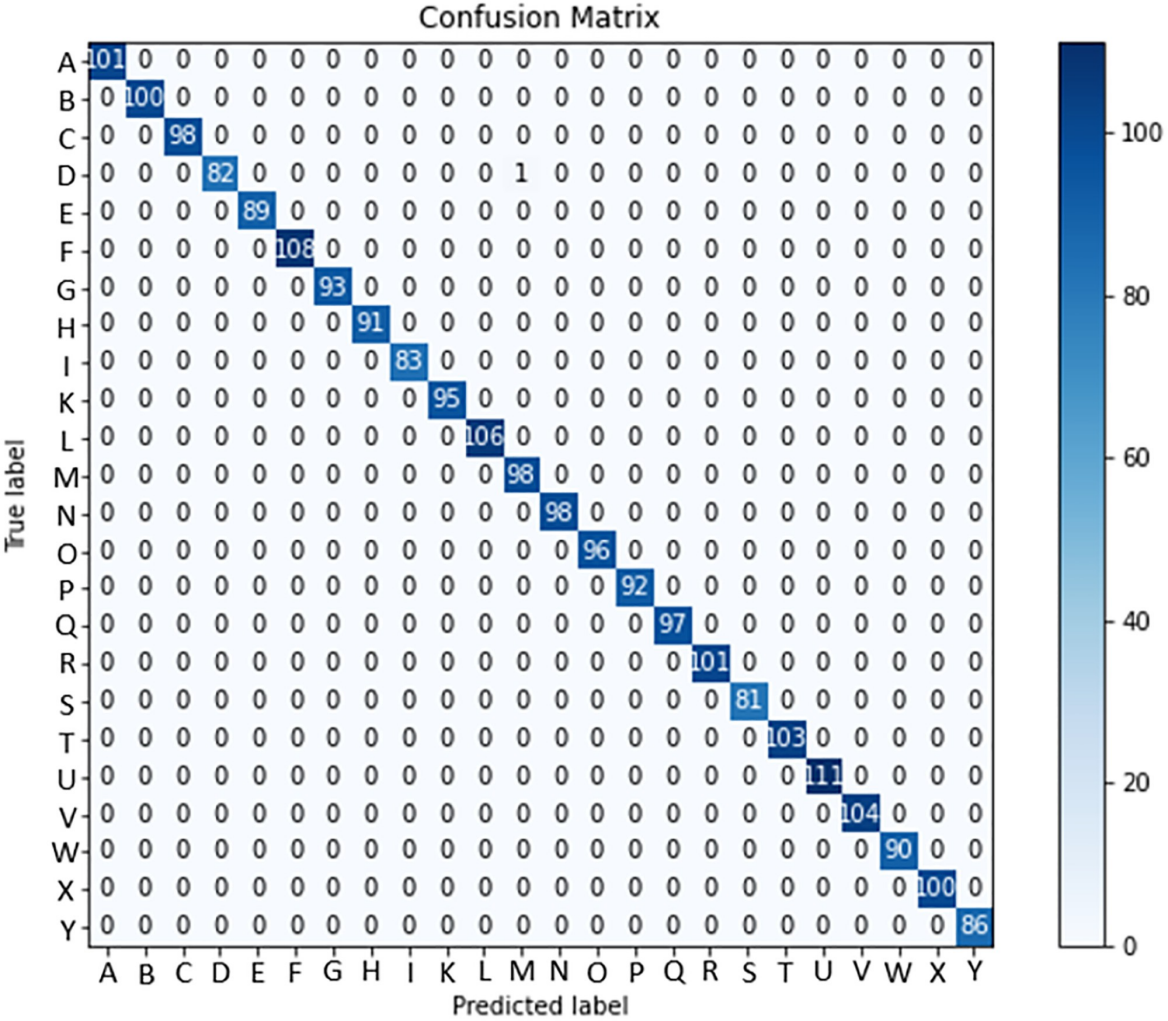
Confusion matrix (ASL Dataset).

**Fig 7:**
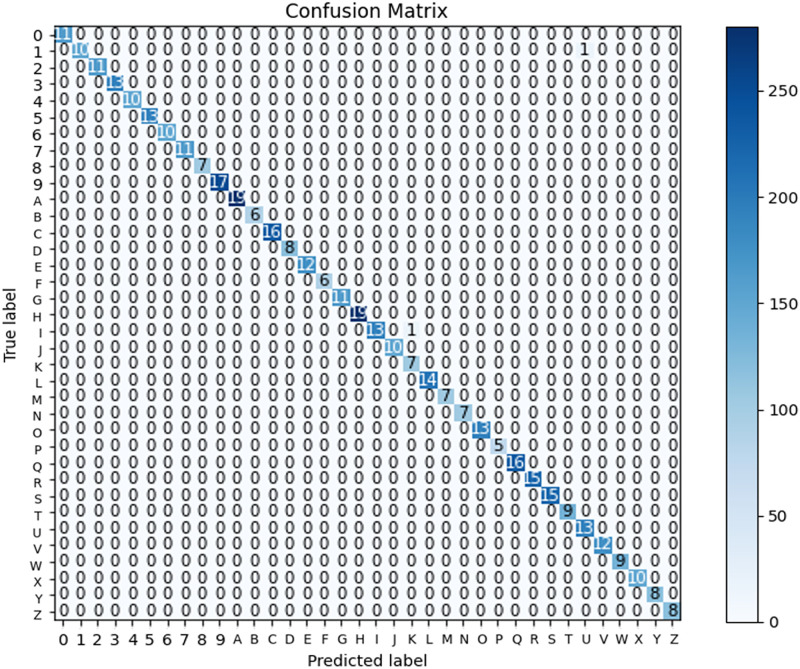
Confusion matrix (ASL with Digits Dataset).

**Fig 8:**
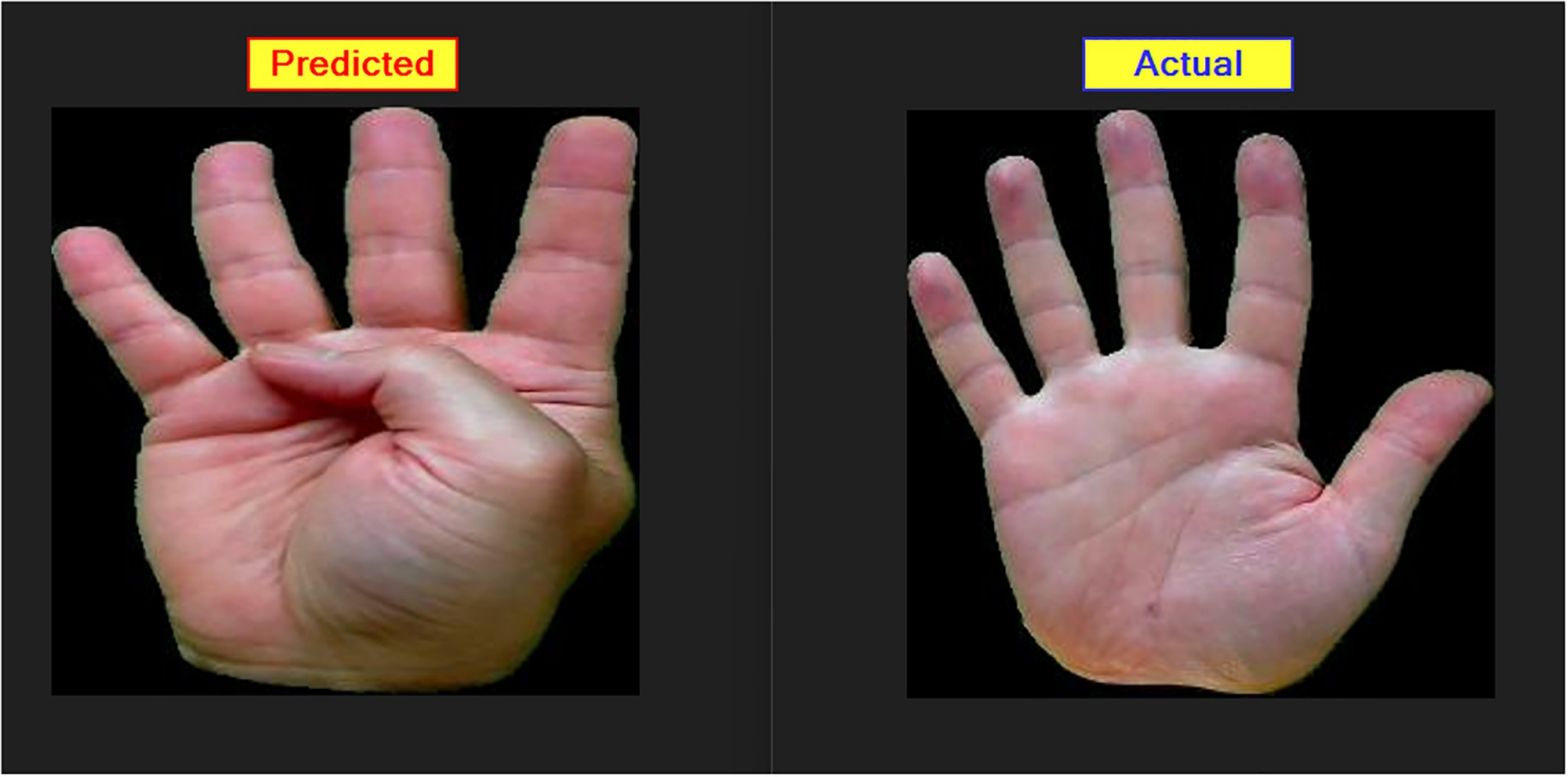
Sample image of digit ‘1’ wrongly predicted as alphabet ‘U’ from ASL with Digits Dataset.

**Fig 9:**
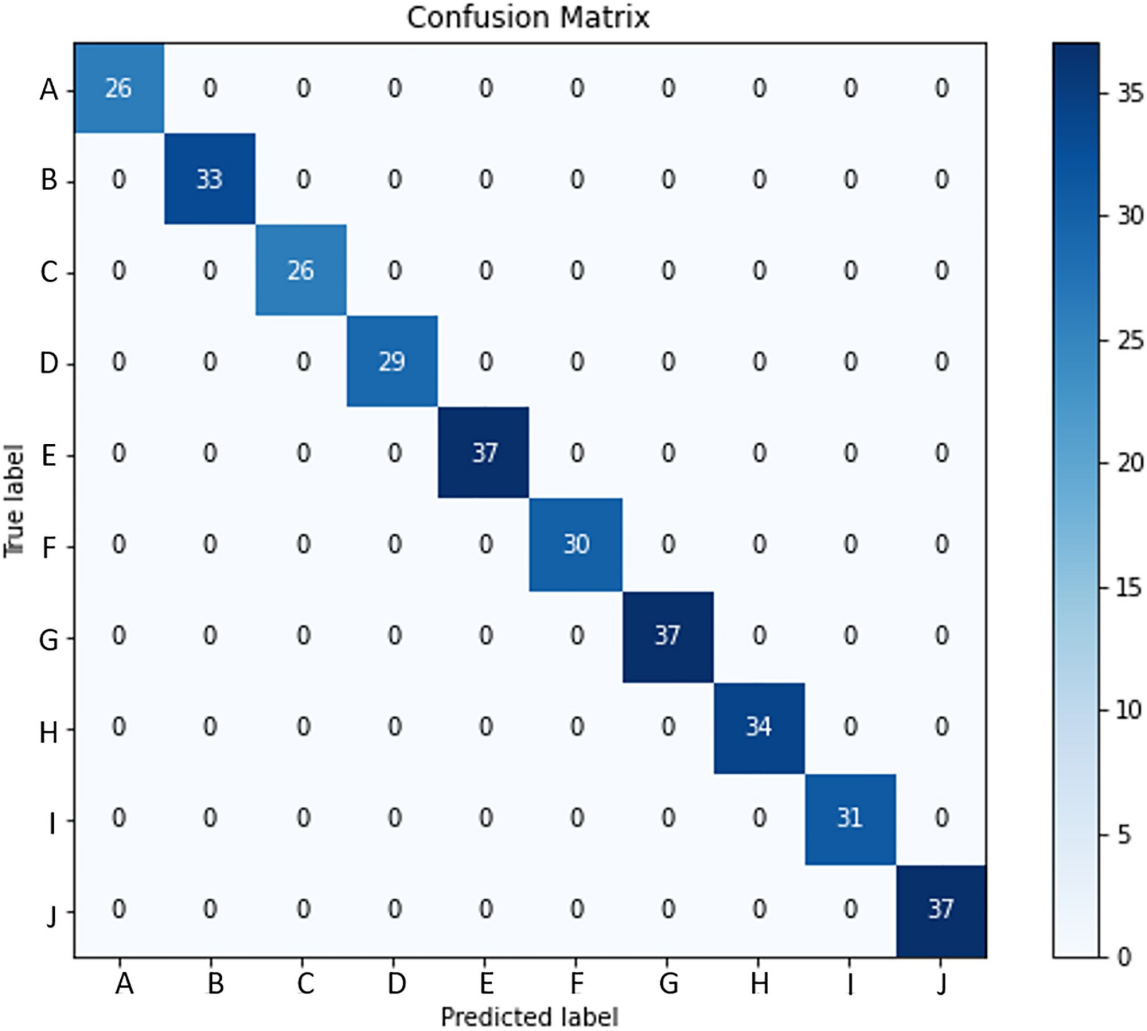
Confusion matrix (NUS Hand Posture Dataset).

In the context of the ASL Dataset, Fig 6 presents the confusion matrix of the LAVRF model, providing a visual representation of the classification performance. Similarly, Fig 7 showcases the confusion matrix of LAVRF on the ASL with Digits Dataset, while Fig 8 depicts an instance of misclassification, where the digit ‘1’ is erroneously classified as the letter ‘U’ due to their high resemblance in gestures. Finally, Fig 9 illustrates the confusion matrix of LAVRF on the NUS Hand Posture Dataset.

## Conclusion and future work

The proposed method, Lightweight Attentive VGG16 with Random Forest (LAVRF), combines a streamlined version of the VGG16 model with an attention mechanism and utilizes a Random Forest classifier for sign language recognition. The Lightweight Attentive VGG16 model preserves key convolutional and max-pooling layers and incorporates attention modules to capture fine-grained details. Attention mechanisms selectively focus on relevant regions, enabling the model to discern subtle differences in hand shapes and movements. The Random Forest classifier leverages the rich representations learned by the Lightweight Attentive VGG16 model. It effectively handles high-dimensional feature spaces, reduces overfitting, and provides robust predictions, especially in the presence of noisy or incomplete data. Hyperparameter optimization using Optuna with hill climbing further enhances the model’s performance by automating the search process and finding optimal hyperparameter configurations. The proposed LAVRF model achieves remarkable performance on three datasets with accuracies of 99.98%, 99.90%, and 100% on the ASL, ASL with Digits, and NUS Hand Posture datasets. Future work can focus on exploring other deep learning architectures, collecting diverse datasets, and integrating the LAVRF model into real-time applications.
